# Progress in Surgery

**Published:** 1897-06-26

**Authors:** 


### Progress in Surgery.

### GENITO-URINARY SURGERY.

(*Continued from page* 199.)

**The Treatment of Stricture of the Urethra,** by excision of the stricture, has not often been resorted to. A case is recorded by Platt,[10] of Manchester, in which a traumatic stricture was excised with satisfactory result. The stricture was recent, and due to injury to the perineum from the wheel of a bicycle. On admission to hospital no instrument, however fine, could be passed, and first Wheelhouse's operation was performed, and a week later half an inch of the urethra (membranous portion) containing the dense cicatricial tissue was excised, and the divided ends of the urethra brought together with sutures over a retained catheter, the perineal wound being drained with iodoform gauze. Although it was necessary for the patient to pass an instrument every now and then, yet he was saved from the troubles of what would no doubt have been a very intractable form of stricture. The case was reported eighteen months after the operation. Platt says that most of the cases operated on in this way have been traumatic, but that the operation has been performed in a few cases of gonorrhœal origin. In this case Wheelhouse's operation would not have preceded excision if the latter had been determined on at an earlier date. The greatest length of urethra which the author considers can be safely removed is about three-quarters of an inch.

Jonas[11] recommends retrograde catheterisation in cases in which, while doing an external urethrotomy for the relief of impermeable urethral stricture, all trace of the urethral lumen is lost, and cannot be found after a prolonged and careful search, and when pressure above the pubes and in the rectum fails to force urine out as a guide to the proximal portion of the urethra. He records four cases out of 11 operations for impermeable stricture in which he resorted to this method.

A very remarkable case of extravasation of urine, following coitus, in a healthy man aged 36, who had no urethral structure, is recorded by Mr. Barwell.[12] Apparently the membranous urethra gave way, a condition of great rarity. The swelling of the scrotum in the morning, after coitus the previous night, was the first symptom. No blood appears to have passed. Patches of local gangrene occurred in the perineum before Mr. Barwell saw the patient, and incisions were made. Recovery took place.

Rupture of the bladder from causes other than traumatism is exceedingly rare. A case of so-called "pathological" rupture of the bladder is recorded[13] by Cuff from the Sheffield General Infirmary. The patient had had symptoms of stricture and recurrent attacks of cystitis for many years. When trying to lift a long, heavy ladder from the wall, he distinctly felt something "give way" in the lower part of his abdomen. There was no pain, however, at the time, and only a feeling of slight discomfort was present during the rest of the day, but next day pain became severe, his temperature rose, and a hypogastric swelling formed. This swelling was explored later on, and was found to consist of connective tissue spaces filled with urine, and in places with pus. A small opening into the bladder was found, but as the patient fortunately recovered, the exact condition of the bladder was not ascertained, but Cuff's theory that a thin walled sacculus had given way during the violent strain seems likely. The rupture was outside the peritoneum. Out of 322 cases of rupture of the bladder from all causes, only seven were due to rupture associated with ulceration or sacculus, and nine to pressure from a retroverted gravid uterus.

The formation of calculi in diverticula of the urethra has been considered by Lieblein.[14] These diverticula are of two kinds, congenital and acquired. The former are very rare—only a dozen instances have been recorded—and are usually situated on the lower part of the urethra between the glans and the root of the penis. The acquired diverticula are situated usually in the membranous portion of the canal, and may be due to the rupture of a retention cyst, blood cyst, or abscess. Calculi may form in any of the diverticula. They extend into the scrotum, and do not obstruct the urethra. Symptoms of urinary obstruction are not marked. A stone formed in the bladder which has passed into the urethra, on the other hand, rapidly sets up retention and surrounding inflammation.

Grünfeld gives[15] the following statistics of foreign bodies in the bladder: Out of 115 cases 68 were in men, and 47 in women. In 41 male cases pieces of catheters and bougies were found in the bladder. In 30 of the female cases foreign bodies slipped into the bladder when introduced by the patient into the urethra, and in 13 concretions formed around silk ligatures which had found their way into the bladder after laparotomy. The author records four cases of his own. In one the foreign body consisted of a fragment of necrosed bone, and in another of a cherry stone which had entered the bladder through a perforation between it and the colon due to the ulceration of malignant growth. An interesting case is recorded[16] by Seiffart of a hairpin in the bladder detected by Röntgen rays. The photographic plate was placed in the vagina, and the exact position of the hairpin ascertained. It had not been pushed into the bladder through the urethra, but had been swallowed in an attempt to produce vomiting.

**Surgery of the Ureter.**—A very valuable paper on uretero-ureteral anastomosis has been written by Wesley Bovée, of Washington.[17] He has collected all the recorded cases of reunion of a divided ureter (11 in number), and publishes one of his own. The anatomy and physiology of the ureter are fully described, and reference is made to the interesting fact, which seems now well established, that urine is propelled along the ureter by ryhthmic contractions of the muscular wall of the duct. In nearly all the cases the ureter was divided accidentally in removal through the abdomen of pelvic tumour (Bovée's own case was the exception, as this was a large abscess, which he removed), and in some cases as much as two inches were removed, but reunion of the divided ends was immediately performed, and not one died from any ureteral complication, though three out of the twelve were fatal. In only one case was there evidence of leakage. Four methods were employed—in three cases by Van Hook's method of lateral implantation, in seven cases by simple transverse end-to-end suture, in one case by oblique end-to-end union by suture, and in another by the insertion of one end into the other. In Van Hook's method the cut end of the distal portion of the ureter is tied, and the end of the proximal portion drawn within it through a longitudinal incision in its wall, by means of sutures passed through the extremity of the proximal end, and brought out through the wall of the distal so as to drag the proximal end into the distal through the longitudinal slit. Van Hook has found this method answer well in experiments on dogs.[18] The method of simply invaginating the upper end into the lower was also successfully practised on dogs by Poggi in Italy in 1887, but other surgeons were not so successful in their experiments. Wesley Bovée objects to the passage of the sutures through the mucous membrane of the ureter in Van Hook's method, as offering an opportunity for urine to escape along the sutures, and he points out that ureteral calculi have formed around ureteral sutures. Bovée considers that the lumen of the divided ureter should always be explored by passing a fine instrument along it before it is reunited, in case stricture is present. If no stricture is present he think it unnecessary to drain the wound. Although normal urine is not likely to do harm if a little escapes into the peritoneum, yet, in case the urine should be septic, he advises that forceps be temporarily applied to the ureter, and he does not think that this temporary pressure will do any damage in so vascular a structure. In the past nephrotomy has frequently been performed when the ureter was injured. This is wholly unnecessary. Ligature of the proximal portion of the ureter would lead to atrophy of the secreting tissue of the kidney from backward pressure. It would be a dangerous procedure unless we were sure the other kidney was healthy, and if the urine were septic or became infected at the time of the operation would be likely to set up suppuration in the kidney. Bovée thinks that implantation into the rectum would be likely to set up septic changes in the kidney from ureteral infection, as Van Hook suggested, and implantation on the skin is attended with the risk of infection to a less degree. If the divided ureter cannot be joined, implantation into the bladder seems the best method to adopt Implantation into the urethra is said to have been successfully performed in one case. After urethral implantation incontinence of urine does not persist, as a well-defined bladder is formed from the urethra, and a sphincter vesicæ from its muscles. As the ureter is elastic, the author considers that as much as three inches can be removed without preventing union of the divided ends. Occasionally the ureter is double, and this condition should be examined for before undertaking a reunion of a divided ureter.

[illegible] **of the Testicle.**—Dr. Cumston, of Boston, publishes[19] a case of that very rare form of epididymitis due not to secondary but to tertiary syphilis. A hard nodule formed in the epididymis, which was supposed to be tuberculous, and castration was considered. The testicle was normal. An admission of primary and secondary syphilis was then obtained, and the swelling cleared up under anti-syphilitic treatment. The author considers that the beginning of tertiary syphilitic epididymitis is generally subacute, the induration is irregular, and often the entire epididymis is invaded, although without adhesions to the testicle. There is a tendency to sclerosis, or the formation of grummata. In secondary epididymitis the disease affects usually both sides, and is generally limited to the head of the epididymis, conditions which are not present in the tertiary form. The onset of gonorrhœal epididymitis is more acute, and the epididymis becomes firmly fixed to the testicle. In tuberculous induration of the epididymis the vas is generally thickened, whereas in tertiary syphilitic epididymitis the cord is normal. Associated diseases of the vas, deferens, or prostate as detected by rectal examination will, of course, be in favour of tuberculous disease. In cases with sinuses examination of the pus for tubercle bacilli may be made. The author calls attention also to the presence of hard tense cysts in the head of the epididymis, which might be considered patches of sclerosis. The prognosis of tertiary syphilitic epididymitis, if anti-syphilitic treatment is undertaken, is very good.

The classical specimen described by Sir James Paget many years ago as a chondroma of the testicle, with infection of the pelvic lymphatics, has been recently microscopically investigated by Dr. Kanthack and Mr. Strangeways Pigg,[20] and they have discovered that the growth is a columnar cancer of the testicle, containing cartilage, but that the lymphatics are not involved, though the growth has extended into the pelvis along the spermatic veins, and entered the inferior vena cava. Secondary growths formed in the lungs.

[10] Medical Chronicle, Feb., 1897. [11] Med. Record, Feb. 6, 1897. [12] Lancet, April 3, 1897. [13] Lancet, Feb. 6, 1897. [14] Beiträge zur Klin. Chirurg. Bd., xvii., Hft. 1., and Epitome Brit. Med. Journal, March 13, 1897. [15] Wien. Med. Blätter, Nov. 26, 1896, and Epitome Brit. Med. Journal, Feb. 6, 1897. [16] Seiffart Centralbl. f. Gynäk, Nov., 1897, and Epitome Brit. Med. Journal, Feb. 6, 1897. [17] Annals of Surgery, Jan., 1897. [18] Journal American Medical Association, 1893, xxi., 911. [19] Annals of Surgery, March, 1897, p. 306. [20] Brit. Med. Journal, Jan. 23, 1897.

